# Identification of PCNA-interacting protein motifs in human DNA polymerase δ

**DOI:** 10.1042/BSR20200602

**Published:** 2020-04-28

**Authors:** Prashant Khandagale, Shweta Thakur, Narottam Acharya

**Affiliations:** Laboratory of Genomic Instability and Diseases, Department of Infectious Disease Biology, Institute of Life Sciences, Bhubaneswar 751023, India

**Keywords:** DNA replication and recombination, PCNA, DNA polymerases

## Abstract

DNA polymerase δ (Polδ) is a highly processive essential replicative DNA polymerase. In humans, the Polδ holoenzyme consists of p125, p50, p68 and p12 subunits and recently, we showed that the p12 subunit exists as a dimer. Extensive biochemical studies suggest that all the subunits of Polδ interact with the processivity factor proliferating cell nuclear antigen (PCNA) to carry out a pivotal role in genomic DNA replication. While PCNA-interacting protein motif (PIP) motifs in p68, p50 and p12 have been mapped, same in p125, the catalytic subunit of the holoenzyme, remains elusive. Therefore, in the present study by using multiple approaches we have conclusively mapped a non-canonical PIP motif from residues _999_VGGLLAFA_1008_ in p125, which binds to the inter-domain-connecting loop (IDCL) of PCNA with high affinity. Collectively, including previous studies, we conclude that similar to *Saccharomyces cerevisiae* Polδ, each of the human Polδ subunits possesses motif to interact with PCNA and significantly contributes toward the processive nature of this replicative DNA polymerase.

## Introduction

Eukaryotic DNA replication requires the concerted action of several enzymes and accessory factors [1–3]. While helicases and DNA polymerases are the key enzyme components; replication factor C (RFC), proliferating cell nuclear antigen (PCNA) and replication protein A (RPA) are the integral structural components of the DNA replication machinery. Thus, these genes are essential for cell survival. Again, interestingly, all these factors are multisubunits. Since, the two strands of DNA are in antiparallel orientation and can only grow in the 5′–3′ direction, the mechanism of DNA duplication for two strands are inherently different. Bacteria and viruses use the same processive DNA polymerase for both lagging and leading strands [4]. However, in eukaryotes, a division of labor among the DNA polymerases has been proposed [1,5]. Extensive genetic studies mostly in yeast suggest that while DNA polymerase δ (Polδ) plays a key role in lagging strand synthesis, Polε plays a similarly important role in leading strand synthesis [6]. Polδ also takes part in leading strand synthesis in certain contexts and positions of the replication fork. For example, in a yeast strain, which lacks Polε catalytic activity, Polδ carries out leading strand synthesis [7]. Also during homologous recombination, re-initiation of replication by DNA Polδ ensures cell survival by replicating both leading and lagging strands simultaneously [8]. Thus, accurate and processive DNA synthesis by Polδ is essential for the preservation of genomic integrity and the suppression of mutagenesis and carcinogenesis [9]. Therefore, it is important to understand the mechanism underlying processive DNA synthesis by Polδ and decipher precise contribution by its subunits in such an activity.

PCNA, the homotrimeric DNA clamp tethers Polδ to the chromosomal DNA and regulates its function during DNA replication, repair and recombination [10,11]. Each PCNA monomer consists of two topologically identical globular domains connected by an inter-domain-connecting loop (IDCL) [12]. The interaction of PCNA-binding proteins with PCNA is mediated by a conserved PCNA-interacting protein motif (PIP) with a consensus sequence Q-x-x-(M/L/I)-x-x-FF-(YY/LY); x being any amino acid [11,13–15]. Previously, we have shown that all three subunits of *Saccharomyces cerevisiae* Polδ; Pol3, Pol31 and Pol32 functionally interact with trimeric PCNA mediated by their PIPs [16]. To achieve higher processivity *in vitro*, all three PIPs are required; however for the cellular function of ScPolδ, along with the PIP motif of ScPol32, one more PIP motif from either Pol3 or Pol31 subunit are essential. Interestingly, human Polδ consists of the catalytic large subunit p125 (PolD1) and accessory subunits p50 (PolD2), p68 (PolD3) and p12 (PolD4) [3,17–18]. Recently, we showed that p12 exists as a dimer both in solution and in the holoenzyme [17]. In several studies, biochemical interaction between each subunit of hPolδ and PCNA has been demonstrated [17–20]. We showed that the oligomerization of p12 at the amino-terminal domain facilitates its interaction with PCNA at the carboxyl-terminal domain. Both the oligomerization and the PIP motifs of p12 have been mapped [17]. The co-crystal structure of a peptide containing the PIP motif of p68 with PCNA has been solved [21]. Similarly, a PIP motif has been identified in the p50 subunit of hPolδ. Far-Western analysis and competition experiment suggested that a 22-mer peptide containing the PIP (_58_LIQMRPFL_65_) of p50 binds PCNA and competes with full-length p50 for binding to PCNA. Since the binding of p50 to PCNA was inhibited by p21, a yet another PIP, it was suggested that the binding sites for both the proteins are mutually exclusive and p21 has a higher affinity than the p50 [22]. However, there are contradicting reports regarding the interaction of PCNA with the catalytic subunit p125 [22,23]. One report suggested that p125 alone could not directly interact with PCNA whereas subsequent reports ruled out such a result by carrying out enzymatic assays. Nevertheless, a physical interaction between p125 and PCNA is yet to be established. Therefore, in the present study, by using multiple approaches, we report direct physical binding of p125 with PCNA, as well as mapping of their corresponding binding sites. We have also reconfirmed the existence of a multisubunit human Polδ’s interaction with PCNA, as has been demonstrated previously in *S. cerevisiae* Polδ.

## Materials and methods

### Generation of various expression constructs for Polδ subunits

Various constructs for the wild-type (WT) or mutant human PCNA for GST-affinity tag purification or yeast two-hybrid analysis or confocal study have been described previously [17]. Most of the constructs of p125, p68, and p12 used in the present study have been described earlier except the site-directed mutagenesis. Site-directed mutagenesis was performed to generate various PIP mutants in p125 and p68. NAP249 (5′-GGT GCT CAC GGG CAA GGC GGG CGG CGC TGC AGC CTT CGC CAA ACG-3′) -NAP250 (5′-CGT TTG GCG AAG GCT GCA GCG CCG CCC GCC TTG CCC GTG AGC ACC-3′) primer pair was used to mutate V999A/L1002A/L1003A in p125 by inverse PCR approach and NAP263 (5′-CCA AAT GAG ACC AGC TGC GGA GAA CCG GGC CCA GC-3′) -NAP264 (5′-GCT GGG CCC GGT TCT CCG CAG CTG GTC TCA TTT GG-3′) primers to mutate F462A/F463A in p68 by PCR. After authenticating their sequence, these ORFs were further subcloned into pGAD424 or any other expression systems.

### Yeast two-hybrid analyses

The yeast two-hybrid analyses were performed using HIS3 as a nutritional reporter system as described before [17,24]. Briefly, the HFY7C yeast strain was transformed with various combinations of the GAL4–AD-PCNA (TRP1) with –BD (LEU2) fusion constructs such as BD-p125, BD-p68, BD-p12, BD-p125 V999A/L1002A/L1003A, BD-p68 F462A/F463A and BD-p12 L104A/Y105A and selected on synthetic dropout media without leucine and tryptophan. To verify interaction, co-transformants were spotted on Leu^–^Trp^–^His^–^ selection media plates and incubated further for 2 days at 30°C before being photographed. Yeast transformants exhibiting growth on plates lacking histidine suggest positive protein–protein interaction.

### Confocal microscopy

Chinese hamster ovary (CHO) cells were seeded on to glass coverslips and cultured in standard cell culture conditions as described before [17]. These cells were co-transfected with green fluorescence protein (GFP)/red fluorescence protein (RFP) fusion constructs of Polδ subunits and using Lipofectamine 3000 transfection kit (Invitrogen). Further, cells were incubated at 37°C with 5% CO_2_ and 95% relative humidity for 48 h. After washings with DPBS, cells were fixed in methanol at −20°C for 20 min and again rinsed with DPBS. The coverslips were mounted using antifade reagent and images were taken with Leica TCS SP5 at 63× objective.

### Protein purifications

All the GST-tagged proteins (p125, p125 V999A/L1002A/L1003A, p68, p68 F462A/F463A and PCNA) were expressed in either *Escherichia coli* BL21 DE3 under T7 promoter or in YRP654 *S. cerevisiae* under Gal4PGK promoter, and purified by affinity chromatography using glutathione sepharose beads (GE Healthcare). Culture conditions and purification methodology were as described before [17].

### Surface plasmon resonance

Interaction of PCNA with p125, p68 and their PIP mutants were monitored using a Bio-Rad XPR 36 surface plasmon resonance (SPR) biosensor instrument as described before [10]. Briefly, ∼5 µg of human PCNA or BSA (∼350 RU) was immobilized on GLC chip by amine coupling method as suggested by the manufacturer’s instructions. Purified Polδ subunits were injected at a concentration ranging from 125 to 2000 nM with running buffer composed of 25 mM HEPES pH 7.5, 10% glycerol, 200 mM sodium acetate pH 7.8, 8 mM magnesium acetate, 1 mM DTT, 0.005% Tween-20 and 0.2 mg/ml BSA, at a flow rate of 50 µl/min for 180 s with a 600-s dissociation phase. Molecular interaction was carried out at 20°C. Further, the dissociation constants were determined, after fitting the association and dissociation curves to a 1:1 (Langmuir)-binding model.

### Co-immunoprecipitation

Co-immunoprecipitation (Co-IP) was carried out using HEK293 cells grown up to 70% confluence in a 10-cm dish containing DMEM supplemented with 10% FBS and 1× penicillin–streptomycin antibiotics. These cells were co-transfected with GFP-PCNA with either FLAG-p125 or FLAG-p125 V999A/L1002A/L1003A or FLAG-p68 or FLAG-p68 F462A/F463A mutant by using Lipofectamine 3000 transfection kit. Cells were grown in a humidified CO_2_ incubator at 37°C. After 48-h growth, cells were harvested, washed thrice with DPBS and immediately resuspended in RIPA buffer (50 mM Tris/HCl pH 8.0, 0.5% Sodium deoxycholate, 1000 mM NaCl, 0.1% SDS, 1 mM EDTA, 1 mM EGTA, 25 mM sodium pyrophosphate, 1 mM β-glycerophosphate, 1 mM sodium orthovanadate and protease inhibitor tablet) and kept for 1 h at 4°C on a rocking platform. Followed by centrifugation at 10000 rpm, the supernatant was collected and protein concentration was determined using the Bradford method. Approximately 500 μg of total protein was incubated overnight with anti-FLAG antibody–conjugated agarose beads. The beads were washed thrice with RIPA buffer and bound proteins were eluted by 40 μl of SDS loading buffer and subjected to 12% SDS/PAGE. The proteins from the gel were transferred to polyvinylidene fluoride (PVDF) membrane, followed by incubation of the membrane with 5% skim milk in PBST for 1 h at room temperature. The blot was washed thrice with PBST and incubated with anti-GFP antibody (1:5000 dilution, cat# ab290 from Abcam) for 2 h at RT. Subsequently, after thorough washings, the membrane was incubated with horseradish peroxidase–conjugated goat anti-rabbit IgG (diluted 1:10000 in PBST, cat# A6154; Sigma–Aldrich), and chemiluminescence was detected with an ECL substrate (cat#32106; Thermo) on the Chemi-Doc MP (Bio-Rad) system.

### Far-Western analysis

WT and PIP motif mutant of p125 proteins were resolved in two 12% SDS/PAGE. One of the gel was developed with Coomassie Blue staining and the second one was transferred to methanol-activated PVDF membrane. The blot was first washed with blocking buffer (BLOTTO: 25 mM Tris/HCl, pH 7.4, 150 mM NaCl, 5 mM KCl, 5% fat-free milk, 1% BSA, 0.05% Tween 20) for 1 h at room temperature. Then, the blot was incubated overnight at 4°C with 10 μg/ml of PCNA in BLOTTO with constant agitation. After thorough washings with BLOTTO, the membrane was incubated with the anti-PCNA antibody (diluted 1:1000, cat#SAB2108448; Sigma–Aldrich) in BLOTTO. Subsequently, the blot was developed with horseradish peroxidase–conjugated goat anti-rabbit IgG (diluted 1:10000 in PBST, cat# A6154; Sigma–Aldrich), and developed as explained earlier [17].

### *In silico* analysis of p125 structure

p125 PIP (_996_TGKVGGLLAFAKR_1008_) and ScPol3 PIP (_993_GSQKGGLMSFIKK_1005_) motifs were used for peptide structure prediction by using PEP-FOLD3 server (http://bioserv.rpbs.univ-paris-diderot.fr/services/PEP-FOLD3/). Further, the generated structural models were aligned with PIP peptide sequences of p12, p21 and p68.

## Results

### Subcellular co-localization of PCNA with hPolδ subunits

**Figure 1:**
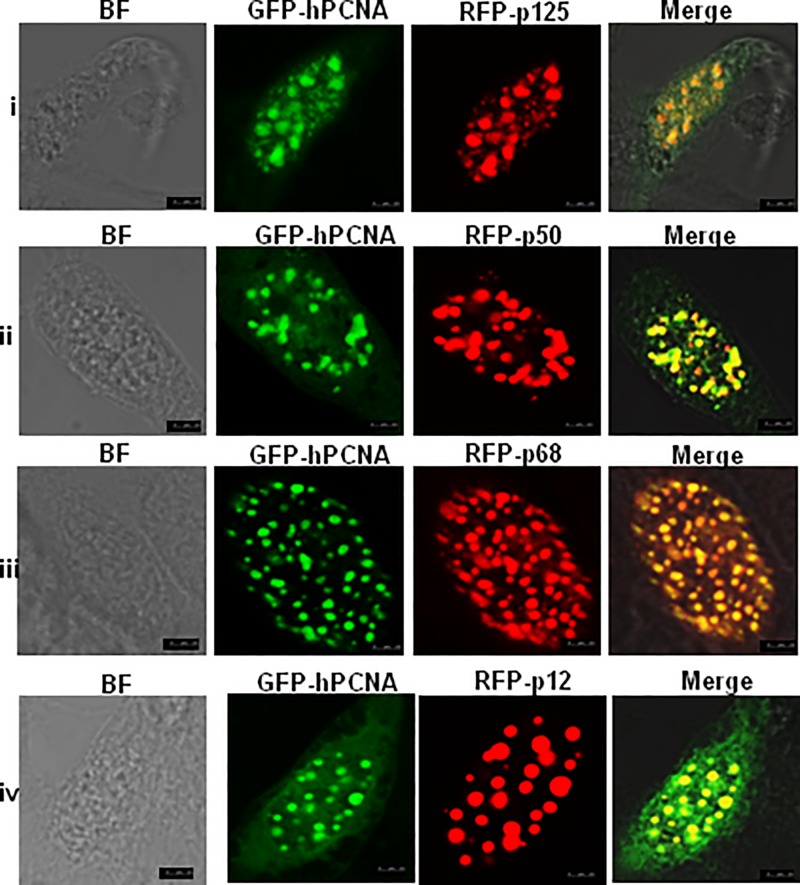
Confocal microscopy analysis of nuclear co-localization of p125/p50 /p68/p12 with PCNA in CHO cells The cells were transiently co-transfected with GFP-PCNA/RFP-p125 (**i**), GFP-PCNA/RFP-p50 (**ii**), GFP-PCNA/RFP-p68 (**iii**), and GFP-PCNA/RFP-p12 (**iv**) constructs. After 48 h, cells were fixed, mounted and protein expression patterns were visualized using CLSM [Leica TCS SP5] at 63× objective. Scale bars represent 25 μm.

In order to confirm the interaction of hPolδ holoenzyme with PCNA, *in vitro* subcellular localization patterns of individual subunits with PCNA in CHO cells were studied using confocal laser scanning microscopy (CLSM). In the nucleus, replisomes are microscopically perceived as distinct foci. These foci are considered as chromosomal DNA-associated large protein complexes functionally active during DNA replication and other DNA transaction processes [25]. Since PCNA functions as a docking platform, DNA polymerases and other accessory factors accumulate with PCNA forming foci [25]. Therefore, to determine PCNA co-localization with hPolδ subunits in CHO cells, these subunits were fused to amino-terminal RFP, and PCNA was tagged to GFP. CHO cells were co-transfected with various RFP-fusion constructs, *viz.* p125-RFP, p50-RFP, p68-RFP, p12-RFP and GFP-PCNA. Co-transfectants were fixed at 48 h post-transfection and observed under the confocal microscope. As depicted in Figure 1, while each of the Polδ subunits developed red foci, PCNA formed distinct green punctates in the nucleus. Subsequent merging of both the foci in the co-transfectants led to the appearance of yellow punctuates. Co-localization of foci revealed that p125 (i), p50 (ii), p68 (iii) and p12 (iv) subunits of hPolδ are physically associated with PCNA and together they developed yellow foci. Since Polδ is a holoenzyme complex, co-localization of p125 subunit with PCNA could be either a direct or indirect interaction that requires further validation.

### Identification of putative PIP motifs in p125 subunit of hPolδ

**Figure 2:**
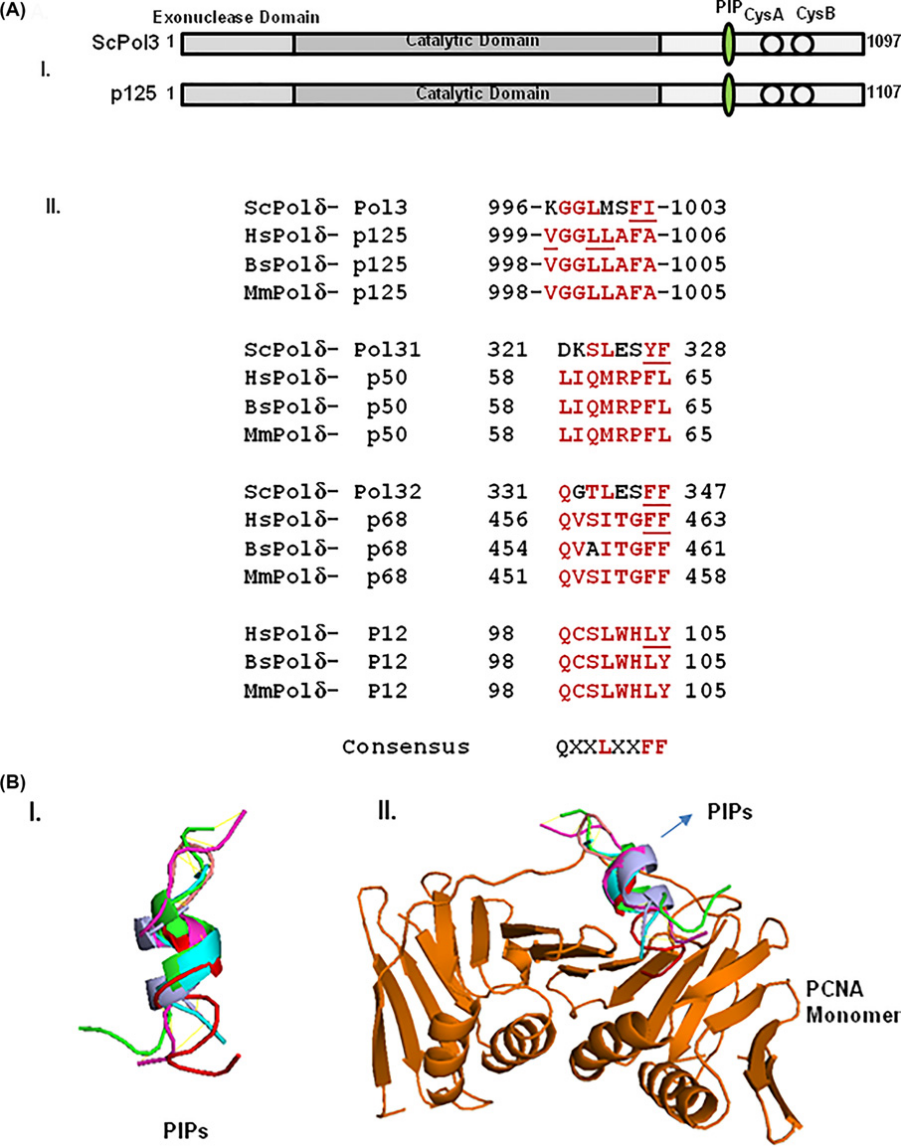
Identification of PIP motifs in Polδ subunits and *in silico* structure prediction (**A**) Ray diagram representation of various domains of Pol3 and p125 and showing the similar location of PIP motifs (**I**). PCNA-interacting motifs in p125, p68, p50 and p12 subunits of Polδ. Amino acid residues in PIP motifs of various Polδ subunits from human (*Homo sapiens*), mouse (*Mus musculus*), bovine (*Bos taurus*) and budding yeast (*S. cerevisiae*) were aligned. Underlined residues have been mutated in various studies (**II**). (**B**) Structure modeling of p125/ScPol3 PIP motifs. Peptide model generated for p125 PIP (_996_TGKVGGLLAFAKR_1008_) and ScPol3 PIP (_993_GSQKGGLMSFIKK_1005_) motifs using PEP-FOLD3 and aligned with already known PIP motifs of p68, p12, and p21 without (**I**) and with PCNA (**II**). PCNA monomer, Pol3, p125, p68, p12, and p21 are shown in orange, cyan, red, green, light blue and purple colors, respectively.

The PIP motif of ScPol3 is located in the vicinity of cysteine-rich metal-binding motifs (CysA and CysB) at the carboxyl-terminal region [16]. At a similar position, we identified a stretch of eight amino acid sequences from 999 to 1006 amino acids in p125 orf (_999_VGGLLAFA_1006_) which shows maximum similarity with the PIP motif in ScPol3 (_996_KGLMSFI_1003_) (Figure 2A,I). The PIP motif amino acid sequence appears to be highly conserved in other vertebrates’ Polδ subunits as well (Figure 2A,II). The crystal structure of the PIP motif of p68 (_456_QVSITGFF_463_) and the modeled structure of the PIP motif of p12 (_98_QCSLWHLY_105_) exhibited remarkable structural conservation and formed 3_10_ helices [17]. However, according to the p50-p68 co-crystal structure, the PIP motif of p50 (_58_LIQMRPFL_65_) is located in the α2 helix of the OB-fold domain and is not involved in any interaction with p68 subunit [26]. Although various structures of Polδ from *S. cerevisiae* are available, the putative PIP motif of Pol3 is not to be visualized, as this portion of the protein is highly unstructured [27,28]. Therefore, we determined the model structures of PIP motifs of Pol3 and p125 and compared them with already confirmed PIP structures. The amino acid stretch of PIP motifs from p125 (_996_TGK**V**GG**LL**AFAKR_1008_) and ScPol3 (_993_GSQKGGLMSFIKK_1005_) were considered for peptide structure prediction by using PEP-FOLD3 server (http://bioserv.rpbs.univ-paris-diderot.fr/services/PEP-FOLD3/) without taking any known template to avoid biased prediction (Figure 2B). Further, the models were validated using the Ramachandran plot, which showed most of the residues in allowed regions. Our structural prediction suggested that as in p12 and p68, both the PIP motifs of catalytic subunits form a typical 3_10_ helix (Figure 2B,I) and superimpose quite nicely with the structure of p68-PCNA co-crystal structure (Figure 2B,II). The model structure also predicts the binding of the p125-PIP motif to the IDCL domain of PCNA (Figure 2B,II).

### Physical interaction of p125 with hPCNA

**Figure 3:**
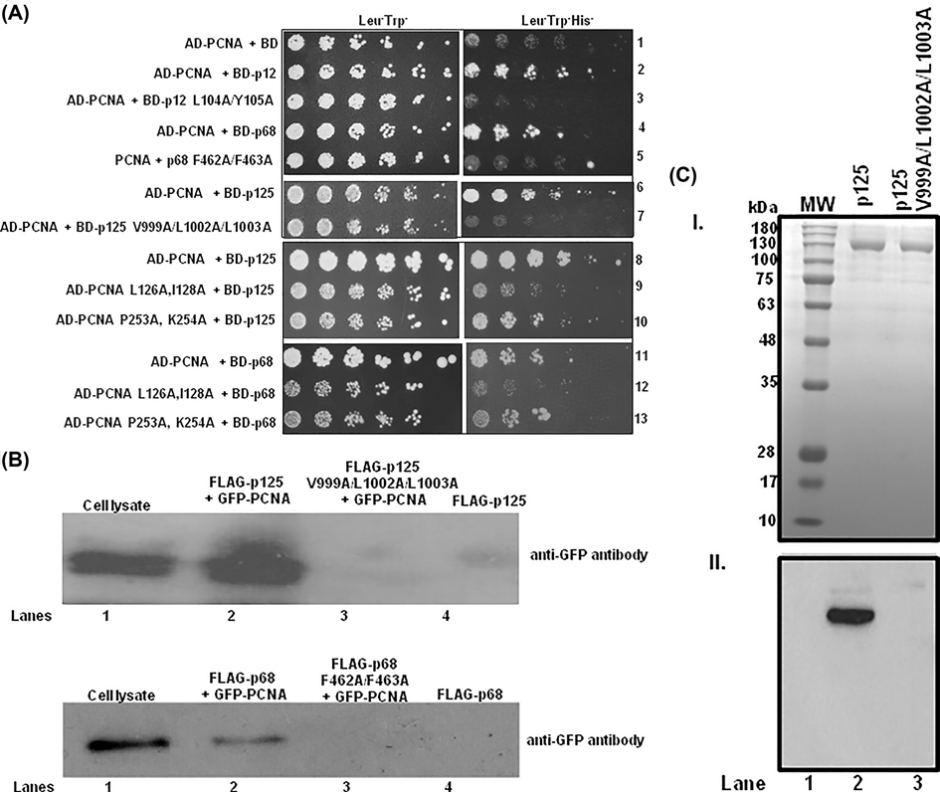
Validation of PIP motifs by yeast two-hybrid and Co-IP (**A**) Yeast two-hybrid analysis showing the interaction of PCNA with WT and putative PIP mutants of p125/p68/p12. HFY7C yeast transformants with various GAL4-AD and BD fusions were selected on SD media plates lacking leucine, tryptophan with and without histidine amino acids. Row 1: AD-PCNA + BD; Row 2: AD-PCNA + BD-p12; Row 3: AD-PCNA + BD- BD-p12 L104A/Y105A; Row 4: AD-PCNA + BD-p68; Row 5: AD-PCNA+ BD-p68 F462A/F463A; Row 6: AD-PCNA + BD-p125, Row 7: AD-PCNA + p125 V999A/L1002A/L1003A; Row 8: AD-PCNA + BD-p125; Row 9: AD-PCNA L126A,I128A + BD-p125; Row 10: AD- PCNA P253A, K254A + BD-p125; Row 11: AD-PCNA + BD-p68; Row 12: AD- PCNA L126A,I128A + BD-p68; and Row 13: AD- PCNA P253A, K254A + BD-p68. (**B**) Co-IP study of p125 and p68 with PCNA. HEK cells co-transfected with plasmids expressing GFP-PCNA and FLAG-p125 or FLAG-p125 PIP mutant or FLAG-p68 or FLAG-p68 PIP mutant were immunoprecipitated using the FLAG epitope and blotted with an anti-GFP antibody. The PIP mutants of p125 and p68 were unable to pull down the GFP-PCNA (lane 3), whereas wild type proteins pulled down PCNA (lane 2). Cell lysate (10%) corresponding to lane 2 was used as an input control (lane 1). FLAG immunobeads of cell lysates expressing FLAG-p125 or FLAG-p68 alone served as negative controls (lane 4). (**C**) Upper panel (**I**) depicting Coomassie Blue-stained gel of p125 proteins resolved in SDS/PAGE; whereas lower panel (**II**) is a far-Western analysis of similar gel. Proteins were transferred from the gel to the membrane, and further, the blot was blocked with PCNA. After washings, bound PCNA was detected by the anti-PCNA antibody. Lane 1, MW, Lane 2, WT p125 and Lane 3, p125 V999A/L1002A/L1003A.

To validate the *in silico* prediction of interaction of the putative PIP motif of p125 with PCNA, *in vivo* interaction analyses were carried out by using yeast two-hybrid and Co-IP (Figure 3). Since the aromatic and hydrophobic residues of PIP motifs bind directly to the IDCL of PCNA and mutation in these residues in ScPol3 abrogated its functional interaction with PCNA, we replaced V999, L1002 and L1003 residues of p125 and F462, F463 of p68 with alanines by site-directed mutagenesis. Both WT and their respective mutants of various subunits of Polδ were fused to the GAL4-binding domain and hPCNA was fused to the GAL4 activation domain. The HFY7C yeast reporter strain was co-transformed with a combination of GAL4 activation and binding domain fusions and transformants were selected on minimal media lacking leucine and tryptophan. The interactions of PCNA with subunits of Polδ in these transformants were analyzed by selecting them on plates lacking histidine (Figure 3A). As reported earlier, also in the present study, we confirmed the interaction between p12 and p68 subunits with PCNA via their respective identified PIP motifs (compare rows 2 and 3, and 4 with 5). We did not observe any growth when the yeast cells only possessed AD-PCNA and BD empty vector (row 1), thus suggesting that positive selection on plate lacking histidine is due to a specific interaction between two fusion proteins. In the same assay, we could also observe the growth of the co-transformant of AD-p125 and BD-PCNA on the media lacking histidine suggesting that p125 also interacts with PCNA. However, the respective p125 PIP mutant (V999A/L1002A/L1003A) did not support the growth of the co-transformant with BD-PCNA (compare rows 6 and 7). This result suggests that _999_VGGLLAFA_1006_, _456_QVSITGFF_463_, and _98_QCSLWHLY_105_ are the bonafide PIP motifs of p125, p68, and p12, respectively. PIP motif of p50 (_58_LIQMRPFL_65_) has been confirmed by another study [22]. Thus, all the four subunits of human Polδ holoenzyme individually interact with PCNA and their respective PIP motifs were now defined.

Next, to validate the physical interaction in a homologous expression system, HEK293 cells were co-transfected with GFP-PCNA and WT or PIP mutants of FLAG-p125 or FLAG-p68 constructs. Using anti-FLAG agarose beads, p125 or p68 proteins were precipitated from the cell lysate. The beads were washed thoroughly and the pull-down of PCNA was detected by probing with anti-GFP antibody (Figure 3B). As depicted in the figure, while PCNA was only co-precipitated with WT p125 and p68 proteins (lane 2), their respective PIP mutants failed to pull down PCNA (lane 3). This result also suggests that both p125 and p68 bind to PCNA in the human cell as well.

Using purified proteins of PCNA, WT and PIP mutant of p125, a far-Western analysis was also carried out (Figure 3C). The PVDF membrane with WT p125 and its mutant was soaked with PCNA during the blocking step, and later the membrane was developed with anti-PCNA antibody. As we could detect binding of PCNA to WT p125 (lane 2) but not to the PIP motif mutant of p125 (lane 3), our result suggested a direct physical interaction between PCNA and p125 mediated by the newly identified PIP motif.

### p125 binds to IDCL of hPCNA

IDCL and of the carboxyl-terminal domain of *S. cerevisiae* PCNA are the key functional interaction regions of yeast replicative DNA polymerases [29]. Mutational analyses suggested that PCNA with I126A, L128A mutations was defective in interaction and DNA replication by ScPolδ, whereas PCNA with P252A, K253A mutations near the carboxyl terminus was defective in physical and functional interaction with Polε. Like PIP motifs of p68 and p12, our predicted model structure also showed p125 PIP binding to the IDCL domain of hPCNA. To confirm the interaction, yeast two-hybrid assay was carried out with p125 or p68 fused to the Gal4 binding domain and two PCNA mutants namely, hpcna-79 and hpcna-90 fused to Gal4 activation domain. hpcna-79 harbors L126A and I128A mutations in IDCL, and hPCNA-90 possesses P253A and K254A mutations in the extreme C-terminal tail of PCNA. As depicted in Figure 3, while the WT hPCNA and hPCNA-90 were able to interact with both p125 and p68 as evident from the growth on SDA plate lacking leucine, uracil and histidine (sectors 8, 10, 11 and 13); hPCNA-79 did not support the survival as it failed to form intact Gal4 by interacting with p125 or p68 (sectors 9 and 12). It suggests that even in humans, Polδ interacts with the IDCL region of PCNA.

### Determination of binding affinities of subunits of hPolδ with PCNA by SPR

**Figure 4:**
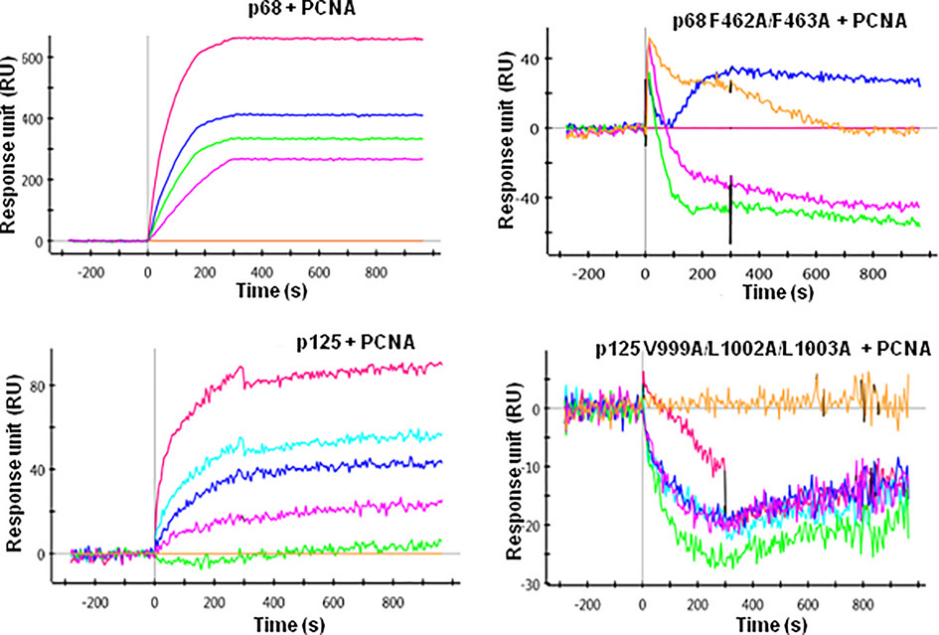
Binding kinetic determination by SPR Kinetic parameters of interaction between p125 or p68 or p125 V999A/L1002A/L1003A or p68 F462A/F463A with PCNA were determined using SPR. Human PCNA was immobilized on GLC chip and various Polδ subunits were passed on it with running buffer at a flow rate of 50 μl/min for 180 s with a 600-s dissociation phase. Further, the dissociation constants were determined, after fitting the association and dissociation curves to a 1:1 (Langmuir)-binding model.

Alignment of PIP motifs in various subunits of Polδ indicated that except PIP motif in p68 other PIP motifs are appeared to be non-canonical that lacks the conserved phenylalanine or aromatic amino acid residues (Figure 2A). The affinity of a PIP motif depends on how accurately the 3_10_ helices fit into the IDCL of PCNA and binding with the neighboring residues [21]. Therefore, we decided to estimate the binding affinities of Polδ subunits with PCNA by using SPR. In this analysis, subunits were passed over the immobilized hPCNA on a GLC-chip (Figure 4). A concentration-dependent increase in the response unit (∼90 RU) was observed when p125 was bound to hPCNA. The interaction of p68 and hPCNA was comparatively stronger than the p125 subunit, and a good increase in refractive index (∼600 RU) and slow dissociation was observed when p68 was bound to PCNA. The *K*_D_ values of p68 and p125 complexes with PCNA was determined to be ∼7 and 100 nM, respectively (Table 1). The assay was repeated with the PIP mutants of p125 and p68. When the mutant proteins were passed over the immobilized PCNA, we did not observe any significant change in the refractive index even when we used ten-fold more than the WT proteins. It suggested that the interaction between WT proteins and PCNA is very specific and requires PIP motifs. In similar assay conditions, we also did not find any significant binding of p12 and p50 with PCNA by SPR analysis (data not shown). However, in our earlier study, we have determined the affinity equilibrium constant of p12-PCNA by isothermal calorimetry (ITC) analysis and is found to be in the range of 146 nM [17].

**Table 1:** Determination of binding kinetic parameters between PCNA and subunits of Polδ

PCNA-analyte	*k*_a_ (M^−1^.s^−1^)	*k*_d_ (s^−1^)	*K*_D_ (M)
**p125**	1.75 × 10^3^	2.0 × 10^−4^	1.14 × 10^−7^
**p125 V999A/L1002A/L1003A**	ND	ND	ND
**p68**	1.92 × 10^4^	1.27 × 10^−4^	6.59 × 10^−9^
**p68 F462A/F463A**	ND	ND	ND

Abbreviation: ND, not determined as no interaction found.

## Discussion

Polδ is the major polymerase involved in DNA synthesis during replication, repair and recombination processes [9]; thus it plays a pivotal role in genome integrity. Apart from the catalytic subunit, the structural subunits of Polδ also play a crucial role in its fidelity and processivity. Several mutations in the mouse and human Polδ subunits have been mapped to be associated with various cancers development [18,30–32]. The objective of the present study was to understand the role of each subunit of hPolδ especially the catalytic subunit in PCNA interaction. Since, hPolδ is a pentameric complex and PCNA is a trimer, the mode of interaction between the two proteins is very complex. Using multiple approaches, we determined the interaction of each subunit with PCNA. By utilizing transiently expressed fluorescently tagged Polδ subunits and PCNA, we used confocal microscopy to study nuclear co-localization of Polδ subunits with PCNA. Our yeast two-hybrid and Co-IP investigations revealed interactions between PCNA and Polδ subunits, *viz.* p125, p68, and p12. Further, the PIP motifs in each of the subunits were mapped. Similar to ScPol3, the PIP motif of p125 is located in the C-terminal domain upstream to the zinc finger motifs and mutations in this motif abrogated PCNA interaction. Luo et al. has already mapped the PIP motif in p50 [22]. Since a peptide derived from p21 inhibits p50 binding to PCNA, it suggested that p50 PIP motif has a weaker affinity toward the IDCL of PCNA than that of p21. When we carefully observed the PIP motif of p50 (_58_LIQMRPFL_65_), we noticed the presence of a basic amino acid which is typically found in APIM (AlkB homolog 2 PCNA-interacting motif), yet another functional PCNA interacting sequence [33]. The co-crystal structure of a peptide possessing the PIP motif of p68 with PCNA has already been solved [21] and this study substantiated the earlier reports. In our earlier study, we have shown that p12 is a dimer in hPolδ, and the dimerization in the N-terminal RKR-motif induces its interaction with PCNA via PIP motif located at the C-terminal domain [17]. Our *in silico* structural modeling studies confirm that these motifs form a typical 3_10_ helix and that stably fits into the hydrophobic pocket in the IDCL of PCNA. Taken all together, we suggest that human Polδ interacts with PCNA via multiple identified PIP motifs, and presence of these multiple PIPs in Polδ holoenzyme will stabilize Polδ’s binding to PCNA that in turn will help in processive DNA replication. Based on our earlier observation in yeast, we propose that during DNA replication p125 and p68 interact with two IDCLs in PCNA trimer and the third PCNA monomer is free to interact with other essential replication factor. When Polδ is not involved in DNA synthesis, other subunits might interact with PCNA and facilitate the recruitment of other DNA polymerase or Okazaki fragment processing enzymes or DNA repair proteins to the primer–template junction. Finally, we suggest that in eukaryotic systems, Polδs establishes a multisite PCNA interaction via its subunits and carry out highly processive DNA synthesis. Considering that improper DNA replication due to altered Polδ interaction with PCNA can affect genomic stability, our study will help to delineate cellular functions of individual subunits of Polδ during various DNA transaction pathways.

## Abbreviations

CHO: Chinese hamster ovary
Co-IP: co-immunoprecipitation
GFP: green fluorescence protein
IDCL: inter-domain-connecting loop
PCNA: proliferating cell nuclear antigen
PIP: PCNA-interacting protein motif
Polδ: DNA polymerase δ
PVDF: polyvinylidene fluoride
RFP: red fluorescence protein
SPR: surface plasmon resonance
WT: wild-type
